# Supplementary material for: Using genetic variation to disentangle the complex relationship between food intake and health outcomes
Source: PLoS Genet. 2022 Jun 2;18(6):e1010162. doi: 10.1371/journal.pgen.1010162 (PMC9162356; doi:10.1371/journal.pgen.1010162)
Supplement: Attachment — Submitted filename: responses to reviewers.docx [file pgen.1010162.s003.docx]

We would like to thank the reviewers for the constructive comments, and we believe we have carefully addressed all their concerns.

We must note that given the reviewers concern and suggestions, we have reviewed deeply the manuscript and have changed some of the methods. This has resulted in rerunning all the analyses from the top.

Main changes include, adding smoking to the exposures which could potentially influence food consumption. To address the problems with weak instrument bias for the group phenotypes we have used a method which can derive a “genetically independent phenotype” GWAS from individual food GWAS summary statistics. This led to new GWAS results for a latent factor capturing the common variance between the items in a given food group and allowed us to select the SNPs which are strong instruments specifically for this latent factor.

These changes have also led to differences in the final MR results. This did not change the main conclusions, but has helped clarifying them and removing some of those which seemed controversial.

We hope that the thoroughly revised, new version of the paper is now suitable for publication.

Following point by point reply to editor’s and reviewer’s comments:

We hope the authors find the thorough review by the reviewers helpful. To be considered responsive it will be critical that the authors respond to all comments. The authors need to describe the assessment toll in detail in the methods (including how the 29 food phenotypes have been assessed) so that the reader can better judge the work. The authors should give particular attention to the issue that they start off with an assessment tool that is known to be biased and error prone. This is likely particularly the case for the UKB given its limited dietary assessment tool that is not a food frequency questionnaire. As part of this a comparison with the 24 hr recall assessment is an excellent idea and needs to be added to the paper for being considered responsive to the reviewers’ comments. Accordingly, the authors should tone down their statements about the superiority of their approach compared to other work.

R. Thank you for this suggestion. The “touchscreen dietary questionnaire” is indeed a food frequency questionnaire including 29 questions about diet and 18 questions about alcohol (PMC5799609). A previous study comparing dietary touchscreen variables with mean intakes from 24h diet recalls in UKB showed that the dietary touchscreen variables, available in the full cohort, reliably rank participants according to intakes of the main food groups. The justification to use FFQ data in our study is because we were interested in long-term information on dietary exposures rather than looking at more granular diet information on foods and drinks consumed during the last 24h that may be not representative of overall dietary intake. Having said that, we share this Referee’s concern that both dietary assessment methods have their own limitations, and that further studies using diet biomarkers, diaries or other technologies could help better understand the relationship between diet and health outcomes.

We have added a reference to this into the materials and methods section.

The authors also need to add a comprehensive discussion to the manuscript about the limitations raised by the different reviewers, particularly about the issues of weak instrumental variable bias.

R: Thank you for this suggestion. We assessed the possibility of weak instrument bias by calculating the F-statistic for each genetic instrument and showed no evidence of such bias. We have added the following information:

Instrument strength was assessed by deriving the F statistic and it was reported for each MR result within table S18.

The authors also missed to point out the excellent work conducted to find objective biomarkers for dietary intake, such as doubly labelled water, urinary nitrogen, minerals, and vitamins that can be used in calibration studies

R. Thank you for this suggestion. While we acknowledge there are reliable biomarkers for dietary intake, it is also worth mentioning that most dietary exposures lack valid biomarkers and that it is usually not feasible to assess dietary biomarkers in large sample sizes.

Why does the # of participants per phenotype varies so dramatically? Does this raising concerns about the reliability of the data?

R. The number of participants vary according to the responses to each questionnaire (i.e., lower number of participants responded to the 24h dietary recalls). Diet data have been used before to investigate the association between diet and the development of metabolic diseases or identify genetic variants for eating preferences. While we agree that there are relevant limitations in the use of self-reported dietary assessment methods that might compromise reliability of dietary data, we do not think the different number of participants responded to each question might create further concerns. We have added the following information in the discussion:

“Further there are differences in the number of participants who responded to the dietary questionnaires, which might represent informative missingness, but its impact is likely modest [https://www.nature.com/articles/s41467-021-21073-y] and its elucidation is beyond the scope of our paper.“

As pointed out by the reviewers the authors need to reconsider the choices of “exposure” variables or causal health related traits they adjusted in the multivariable MR analysis. Related to this it is not clear how it is possible to investigate the association between foods and BMI if as part of the correction all SNPs that show associations between BMI and the foods were excluded. The same question holds for other “exposure” variables or causal health related traits they adjusted in the multivariable MR analysis.

R. We thank editor for the comment. We have chosen a reasonable wide range of potential exposures. This does not obviously cover all possible exposure influencing food choices which would be impossible to achieve. We believe however that we have been the first to ever take into consideration so many parameters and they do include many common risk factors, hence we believe that our effort is already a tremendous step to move the field forward.

Regarding the second point, we realise that our approach is quite complex and our description was not sufficiently detailed, which we have improved in the revised version. To respond to the Editor’s question, we do not remove SNPs which show association to BMI or other exposures, but we remove SNPs where we have evidence that part of the effect is mediated through a confounder or the exposure. That is possible because even in cases where reverse causality exists if a genetic variant has a direct effect on the exposure this will be much larger than the effect the same SNP has on the exposure through reverse causality. Let’s imagine we have a SNP which acts on “beef” directly with an effect alpha, beef then has an effect of beta on BMI and BMI has a causal effect of gamma on Beef reporting/consumption.

Hence, the total effect we observe on a GWAS would be:

Alpha + alpha * beta * gamma.

$SNPtot=\alpha+\alpha*\beta*\gamma$

If we consider all standardised the reverse causality term $\alpha*\beta*\gamma$ becomes very small very quickly and thus $\alpha$ >> $\alpha*\beta*\gamma$. This means that in the case of a direct effect only SNP $\alpha\approx\alpha+\alpha*\beta*\gamma$ and thus the ratio between the corrected effect and the original one will be close to 1.

The paper does not describe well what results are derived from the UKB vs the other 2 UK cohorts included. This needs to be clearly described in the main text (not only in one of the many supplemental sections)

R. The two additional cohorts were used only for replicating the results from the main analysis and thus no other results are based on it. We explain it better in the manuscript.

As data on smoking are available the authors should investigate if the association between unhealthy diet and lung cancer is truly confounded by smoking or not.

R. Thanks for this suggestion. We have now included smoking as a potential confounding variable and effects are now corrected for it. As a result, the association between genetically predicted overall unhealthy diet and lung cancer is no longer significant.

Reviewer's Responses to Questions

**Comments to the Authors:
Please note here if the review is uploaded as an attachment.**

Reviewer #1: The authors write: "we develop a statistical genetics framework that enables us, to directly assess the impact of food choices on health outcomes." No genetic analysis can ever do this, let alone the one implemented in this study.

The authors write in the Abstract that: "that the biases which affect observational studies extend also to GWAS, genetic correlations and causal inference through genetics, which can be corrected by applying our methods." This is not actually demonstrated in the study. The authors simply apply some methods (which are mix of previously described approaches) and generate some results, but we don't know whether they correct any previous biases.

The authors are overly critical of previous studies in the introduction without any supporting citations or solid evidence for their claims, yet then rely on self-reported data themselves.

We appreciate Reviewer #1 thoughtful suggestions. Following these suggestions, we have lowered the tone of our study and provided a more balanced view of our approach and previous studies.

In essence, the paper boils down to multivariable Mendelian randomization. However, the description is incredibly confusing and the authors seem to constantly mix up mediation and genetic confounding (which cannot be distinguished through statistical analysis alone - biological insight is required).

R. We apologize for any confusion. We have amended the text accordingly to clarify that we refer to cofounding due to the effect of traits that mediate the effect of the SNP on the outcome.

The choice of outcomes is poorly defined - why not also look at osteoarthritis, stroke, peripheral arterial disease, kidney disease, liver disease etc, which are also affected by dietary choices?

R. Thanks for this suggestion. The extensive number of outcomes included in this study was reported in table S17. The rationale not to include some of the suggested outcomes is because were limited in choice to large GWAS which did not include UK biobank. The use of outcomes with UK Biobank samples would create sample overlap and bias in the causal effect estimation.

No attempt is made to address weak instrument bias in a multivariable Mendelian randomization setting. This will likely be a major limitation, as the variants explain a small proportion of the variance in the exposures and their associations with different dietary choices are correlated.

R. We would like to clarify that we conducted multivariable MR only to obtain the direct effects and not for the effect of the food exposure on the outcomes. All our analyses from single foods to PC traits are univariate. We changed our approach to variable selection to avoid the possibility of including weak instruments for the Principal Component trait.

We have selected to use only strong instruments and have reported the F statistics which is always >30 but often closer to 40 or above. We thus should not suffer from weak instrument bias. F-statistic for each of the MR analysis was added to the full MR results in Table S19.

We have however also included MR RAPS (methods section line 351) amongst the sensitivity analyses which is robust to weak instrument bias.

Neither does the paper seem to have been properly proofread. For example, on line 426 it says "[citation error]".

R. We have corrected the citation.

Reviewer #2: This is an exciting piece of research that aims to disentangle the association between diet and health outcomes. The authors have build a thorough analytical pipeline to GWAS dietary factors in UK Biobank, replicate findings in independent cohorts and apply novel design ideas within the Mendelian randomization (MR) framework with an aim to achieve unbiased MR estimates for diet and health outcomes. There is dearth of MR findings for dietary intake and health outcomes, as it is difficult to identify robust and non-pleiotropic genetic instruments for dietary intake (except for alcohol, coffee and dairy intake), and the authors have tried to address this limitation of previous research. They should be commended for their approach, but there are some issues I would like to raise that could partially invalidate some of the approaches.

1) The UKBB study has not used a FFQ for dietary assessment, instead there was a touchscreen diet questionnaire offered to the totality of participants. This small but important misconception should be corrected in the Introduction. As a consequence, the likelihood of measurement error in the dietary assessment is higher compared to the use of a more established method of dietary assessment like the FFQ. In addition, only a relatively small range of dietary intakes were assessed with the current questionnaire that might bias the definition of healthy or unhealthy diet pattern that the authors used. Some further discussion on these issues is required. In addition, ~140K participants have completed at least 1 of the 4 web-based 24h dietary assessment post-recruitment, and it would be nice to see a sensitivity analysis with these data.

R. Thank you for the overall positive evaluation of our study. We would like to mention that the touchscreen questionnaire is a food frequency questionnaire that asked twenty-nine questions about diet, most of which gathered information about the average frequency of consumption of main foods and food groups over the past year (PMC5799609).

We concur with the reviewer that It may not be the same type of long and extensive questionnaire used by other large studies (i.e. EPIC) but it remains a food frequency questionnaire. The validity of the questionnaire used has been examined by Bradbury et al 2018 (as suggested by the reviewer) though comparing the results with the 24h recall. The results indicate that “comparison with the mean intakes from the 24-h dietary assessments showed that the touchscreen variables reliably rank participants according to the intake of main foods and food groups.”

2) The authors have performed MR analyses of several "exposures" (i.e. BMI, lipids, BP, education, T2D and CHD) in relation to dietary intakes and they have used these results via MR mediation analyses to estimate the association of diet to health outcomes not mediated via one of these exposures in an attempt to limit horizontal pleiotropy. This is a smart idea! However, there are couple of additional exposures that may be important to consider further such as smoking and physical activity. In addition, it was not clear in the manuscript whether the authors have performed bidirectional MR analyses between the dietary intakes and the "exposures". In the presence of a bidirectional association, the approach would likely lead to collider bias or adjust out the complete genetic variants to diet association. More formal evaluation and discussion of the assumptions of mediation analysis is required.

R. We have rerun all the analyses in the paper making several changes. Amongst others, we have added smoking to the list of exposures for the initial multivariable MR. The reviewer is correct, and using corrected betas could (and does) lead to collider bias. That is the reason behind the choice of using the mediation analysis use only the SNPs which show little sign of mediation and use their uncorrected effect sizes in the MR. This not only avoids collider bias but also the induced correlation between the exposure and outcome GWAS introduced by the use of the outcome to estimate the effect of all SNPs on it. Let’s imagine that we have for simplicity only one exposure we want to correct our GWAs by, for example we want to correct the effect of BMI on the Beef consumption GWAS. We first use MR to estimate the effect of BMI->Beef consumption. We then multiply the effect of each SNP on BMI by the MR effect and subtract it from the Beef GWAS. This also includes the SNPs which have true effect on BMI of 0 but practically do have an estimate. Applying this genome-wide induces a underlying correlation between the betas and may bias the final MR results.

3) The authors used a stepwise pruning for heterogeneity for their definition of instrumental variables, which is an interesting approach but I would be hesitant to base this process only on statistics (i.e. the p-value for heterogeneity) and would like to see biological knowledge of gene function to take a central role in the process. The authors should check the association of their final selected IVs with any secondary traits and comment on residual horizontal pleiotropy.

R. While we have changed approach and used MR Radial instead of the step wise selection, thus remains a purely statistical approach. Given that we have performed ~5000 MR analyses in this study, checking the biological knowledge and gene function manually is impossible. Moreover, although we do have biological knowledge for some of the exposures for most of them this is not the case and it is not easy to identify for each of them a biologically validated anchor. In addition, we saw very little heterogeneity (low Q-statistic) when using multiple instruments for each exposure, which is a reasonable indicator of lack of horizontal pleiotropy, supporting instrument validity.

4) The authors defined locus definition and independence based on physical distance (e.g. 250kb). Did they consider also LD structure in different genomic areas?

R. Our approach implicitly considers LD as we have not considered distance from the top SNPs to define loci, rather we consider the peak shape. In fact, if we consider usual association peak shape, we have a series of SNPs which are correlated to the main signal and thus “come up” with it. By defining the peak boundaries as the lack of any SNP with p<1e-5 within 250kb from others implicitly implies LD. It is because the association test statistic (which is a monotonic function of the association P-value) between the lead SNP and other SNPs is proportional to their LD.

5) The final MR results of dietary intakes in relation to health outcomes are impressive, and some but not all associations seem to coincide with the hypothesized directions for the associations. However, most of the associations do not survive the multiple comparison p-value threshold and even less survive the sensitivity MR analyses, which the authors should comment clearer upon in the Results text. Could the authors discuss the potential reasons for this? Does their method lead to lower statistical power or to bias in some circumstances when adjusting for exposures acting as colliders?

R. The new results we present are more in line with the hypothesised causal association than before they were before. We have improved the way we select the SNPs for composite traits and have kept only SNPs with high imputation scores for all analyses which should reduce bias due to bad imputation. This has led to a reduced number of results but also to sturdier ones as indicated by the lack of residual heterogeneity for all of them.

Given that we use the original effect sizes collider bias should not affect our results.

The drawback of using IVs with clear evidence of only direct effect is the loss of statistical power due to the reduced explained variance by the selected instruments. Future developments could address this issue by finding the optimal trade-off between bias and power, e.g. by up-/down-weighting instruments as a function of their estimated validity, instead of strict exclusion.

Reviewer #3: General comments:
This is an interesting work on a very relevant topic, whether Mendellian Randomization (MR) analysis can help circumvent some of the chronic limitations of nutritional epidemiology when relating the intake(s) of specific foods, as well as patterns of foods, to health outcomes. The study used genetic and dietary data from the UKB, as well as data from several available datasets, to identify genetic variants to be used as instrumental variables in etiological models.
Nevertheless, several important aspects of the current approach are not fully convincing, nor several details of the employed methodology are sufficiently clearly described. As a result, the description of the methodology reads very confusing.
In addition, although the study attempts to address weaknesses of nutritional epidemiology like confounding, reverse causation and exposure measurement errors, within the genetic framework employed, genetic instrumental variables for food intakes are estimates in models where dietary assessments by means of food frequency questionnaires were used. It is well known that these measurements are prone to exposure misclassification, potentially leading to biased identification of relevant genetic variants. These limitations are not even addressed in the current version of the manuscript.

R. We have added reference to previous work comparing the FFQ in UKB to the 24h recall questionnaires which shows that “comparison with the mean intakes from the 24-h dietary assessments showed that the touchscreen variables reliably rank participants according to the intake of main foods and food groups.” Therefore, we believe that this is reasonably reliable, at least compared to other questionnaire-based methods.
We realise that the method is complex and have further improved the text, we hope the changes have made it clearer.

We believe that we have created a statistical approach which is capable, to some extent, to distinguish SNPs which have a direct effect on the food exposure from those biased by other factors, so addressing reporting biases, reverse causation and confounding is at the very heart of the manuscript. Of course this procedure is not perfect and misclassification and measurement error can be still present, we have included the a sentence in the discussion to reflect this.

Detailed comments:
Abstract, line 48: “determinants of dietary intake” – what dietary intake, how many food groups, how were they chosen?

R. We used all the items available in the dietary touchscreen questionnaire in UKB. The choice of the items was made by the UK biobank team to capture the consumption of major food groups.

Line 50-51: the text reads very optimistic in describing the approach but no details whatsoever are provided to enable the reader understand the novelty and the relevance of the work. In line 53, the text mentions “our work” but again no elements are given to capture the importance of the study. Overall, the abstract is improvable. It reads rather vague and does not release elements necessary to judge the relevance of the study.

R. We have corrected the abstract according to the reviewer’s suggestions.

Line 56, the text reads “…and several other risk factors”. Should other morbid condition or health outcomes be mentioned instead?

R. We have corrected the text to state “other risk factors and health outcomes”

Line 114: the text is very technical, though some details related to the relevance of the use of the “ldsc” command should be disclosed.

R. We have added the reference to the ldscores used and HapMap 3

Lines 116-117: the reasoning leading to the choice of using genome-wise (statistical) significance threshold equal to 10xE-8 in the study is not explained clearly.

R. Bonferroni correction consists of dividing the selected significance threshold by the number of independent statistical tests. Given that we identified 5 independent food groups we divided the GW threshold by 5. We have explained this better in the text.

Lines 120-121: The text does not read appropriate.

R. We have corrected the text.

Lines 123-124: MR analysis is NOT conducted to measure (estimate) the effect of health outcomes on food consumption. Rather the other way round.

R. We have first performed an MR analysis to measure the effect of health outcomes on food consumption (reporting). These causal effects are then used to estimate the direct effect of each SNP on the food of interest in order to perform another MR analysis where the food consumption is the exposure and health-related measures serve as outcome. The use of the first MR analysis is to help excluding potentially invalid instruments in the central food->disease MR analysis.

Lines 124-125: “foods for which nutritional advice is given”. This is an important information, but it does not clarify what criteria were used to pick up the chosen list of food items this study focused on.

R. The food items were chosen based on what was available in the FFQ in UK biobank. Unfortunately, the questionnaire is not large, and it doesn’t cover all foods.

Line 128, “we included educational attainment” – Where?

R. Educational attainment was included as exposure in MR of the factors influencing food consumption. We have clarified this further in the text.

Line 131: SNP were included if p<5 x 10E-8 and r2<0.001. Up above the criteria seemed to be 10E-8, not 5 x 10E-8. Also SNP should be selected if r2 was greater than 0.001, not lower.

R. In the MR analysis the reviewer is referring to, the reported food consumption was the outcome and not the exposure, hence the 5x10-8 threshold, mentioned by the reviewer, was applied to the instruments associated with the potential confounders (not the food items), coming from an independent GWAS study.

Lines 139-149: The text does not read correct, nor the MR description is appropriate.

R. We have changed the text to clarify the methods.

Line 150: what is bGWAS?

R. bGWAS in an R package for Bayesian genome-wide association studies. We changed the text to clarify what bGWAS is and provided reference for it.

Lines 150-159: The description of the MR methodology used in the study is very confusing.

R. We have clarified the text.

Lines 120-121: The text does not read appropriate throughout page 7. Statements like the ones in lines 152-154 are really obscure. The CUR approach should be referenced. The analysis moved from univariate to multivariable methodology, but the various steps are not clearly outlined.
There is a massive amount of information provided as a supplementary material and Tables. It is not ideal to exhaustively capture several nuances of the study.

R. The CUR method is introduced in this paper so there is no previous reference available. We have validated it through extensive simulations in the supplementary material. We apologise that we were forced to move a large chunk of the results to the supplementary materials otherwise the main manuscript would have become far too long. It was a difficult choice what to include in the main text to contain the important results for most readers.

Reviewer #4: This is a comprehensive analysis and interesting paper that assesses the utility of using genetic data to unravel the complex relationship between dietary intakes and health outcomes. Some comments:
• Smoking seems like an omission from the analysis as it is well established that smoking influences dietary intakes. The authors included educational attainment as it is likely to affect food consumption. What was the rationale for not including smoking?

R. There are many traits that influence food choices and consumption. We have included many we thought to be important and obviously many other exists. We have now included also smoking.

• Within the UK Biobank, dietary intake has been assessed 4 times during the follow-up period for a subset of participants. How do the genetic variants related to dietary intake identified using the baseline/enrolment (2006-2010) data associate with self-reported intake during the follow-up period assessments (2012-2013; 2014+; 2019+)?

R. We do have not looked at this and we believe that it is beyond this specific work which is already quite extensive. Another issue is sample size where only 70 thousand samples have 2 data points and very few all 4. It is certainly a good idea for a follow up study.

• The authors mention in the results text that contrary to their expectations, the BMI-raising allele was consistently associated with lower reported consumption of energy-dense foods such as meat or fat, and higher reported intake of low-calorie foods. This observation should be discussed further. Do the authors have a possible explanation for this finding?

R. We believe that this is due to either reporting bias or people genuinely try to improve their diets upon weight-gain. This observation has initiated the project behind this paper.

• Did the authors consider multivariable MR analyses for the full MR results (Table S18)? One of the key findings was a positive effect estimate for alcohol consumption on lung adenocarcinoma risk. It would be good to see multivariable MR analyses adjusted for lifetime smoking behaviour.

R. We agree with the reviewer although in this specific case the association between alcohol consumption and smoking is not significant anymore. This is likely due to the more stringent approach we have adopted for the new version of the paper.

• Why did the authors use education as a proxy of SES when Townsend deprivation index may be a more appropriate indicator?

R. We needed an independent GWAs to run MR using UK biobank as outcome. The only large GWAS of SES included UK biobank and was thus unusable in an MR framework. We thus turned to EA as the next best option.

• Did the authors consider validation of the 2-sample MR results in other studies/populations (e.g., FinnGen, Biobank Japan)?

R. Again this is a great idea for a follow up study but replication of results in other studies goes beyond the scope of this paper, especially because diet (and dietary items) may be too country-specific to be generalizable.

• The paper although generally clearly written has several grammatical errors and inconsistent formatting throughout. A thorough edit of the paper is required.

R: Thanks for this suggestion we have reviewed the grammar and formatting.
