# Supplementary material for: Using genetic variation to disentangle the complex relationship between food intake and health outcomes
Source: PLoS Genet. 2022 Jun 2;18(6):e1010162. doi: 10.1371/journal.pgen.1010162 (PMC9162356; doi:10.1371/journal.pgen.1010162)
Supplement: Attachment — Submitted filename: Response to reviewers.docx [file pgen.1010162.s004.docx]

We would like to thank the reviewer and editor for the comments. Following point by point responses.

Editor comment:

Comparing one dietary assessment tool (FFQ) relying on self-report with another dietary assessment tool (24-hr recall) relying on self-report is not proof that the assessment in valid. It would be different if measurements like doubly labelled water or 24 hr urinary nitrogen would be available. Accordingly, it is critical that the authors address these weaknesses and the potential that the exposure misclassification can lead to biased identification of relevant genetic variants and the possible consequences in the discussion. As part of this the authors need to add a discussion on the use of objective biomarkers for dietary intake, such as doubly labelled water, urinary nitrogen, minerals, and vitamins that can be used in calibration studies.

R. We have added a paragraph in the discussion to emphasise this issue.

It seems like a strong assumption that the impact of the sizable missingness in the response to the dietary questionnaire is likely modest. This statement needs to be revised and the range of % missing should be added.

R. The number of missing is relatively high specifically on alcohol traits. This is due to the choice of limiting the analyses to people who regularly drink alcohol. In UKB alcohol consumption is relatively low with 40-60% of people reporting drinking no alcohol at all. We decided to limit the analyses to drinkers because, because the population is older and more educated, people are limiting their alcohol consumption. A similar choice was made for coffee were stratifying by type required to limit the analysis to the people who reported drinking that type of coffee. We have added this detail in the description of the phenotype to make the point clearer and have added this weakness to the Discussion.

Reviewer #4: This is a really interesting study with a clever approach to study the role of dietary intakes on health outcomes. The authors are to be commended for the extensive work undertaken for the revised manuscript. I have the following comments:
• The study design is quite complex. Please could the main text include a figure summarising the different aspects of the study design.

R. We have added a figure to the paper.

• Why was smoking not considered as a health related trait to be assessed in relation with food intake (while education/SES is)? The rationale for the list of exposures could be better described. Smoking is often associated with unhealthy diet pattern but also with weight loss. Could the authors conduct a sensitivity analysis to examine the effect of smoking on food intake? It is very plausible that smoking would influence the intake of food traits and warrant inclusion.

R. Smoking was not initially included in the univariate MR analysis however it was included in the bGWAS correction. This is reported in figure S2 and line 201. We realise that the version of the table S6 was incorrect as it did not report the smoking traits.

• One of the authors recently published a nice editorial (PMID: 34980908) on the challenges of using genetics in nutritional epidemiological studies. One of the challenges rightly mentioned is taking into account how dietary intakes can change over the lifecourse. The authors should undertake a sensitivity analysis on this using UK Biobank data. Approximately 49,000 UKB participants had repeat diet assessment during an imaging visit ~8+ years after the baseline information was collected (<https://biobank.ndph.ox.ac.uk/showcase/field.cgi?id=1369>). How stable/consistent were participants dietary intakes between the baseline and imaging visits? Are a similar pattern of genetic signals found when a GWAS of food intake measured during the imaging visit diet is conducted (n=~49,000 participants)?

R. This is a very interesting question which affects MR studies in general. Restricting our analyses to participants which have responded to the questionnaire at multiple time points will introduce a strong selection bias and it is unclear whether this bias would be compensated by the gain in estimation accuracy. Furthermore, such analysis would be heavily underpowered for any follow-up time point and we have virtually no statistical power to detect differences in effect sizes unless those substantially differ. However, we believe the time-varying causal effects are worth pursuing and their methodological foundations are subject to current investigations [https://www.medrxiv.org/content/10.1101/2022.01.04.22268740v1]. Given the already result-dense nature of our current manuscript, we feel that such investigations are out of the scope of this work.

• There remain several typos throughout the paper and also in the Shiny app. For example, line 72 – “Mendellian”; line 91 – “Hell et al”; throughout – words capitalized mid-sentence. Please ensure that a careful editorial review is undertaken.

R. We have thoroughly revised the text and corrected the typos.
