# Supplementary material for: Using genetic variation to disentangle the complex relationship between food intake and health outcomes
Source: PLoS Genet. 2022 Jun 2;18(6):e1010162. doi: 10.1371/journal.pgen.1010162 (PMC9162356; doi:10.1371/journal.pgen.1010162)
Supplement: Attachment — Submitted filename: Response to reviewers.docx [file pgen.1010162.s005.docx]

We would like to thank the reviewer and editor for the comments. Following point by point responses.

Editor comment:

It is not clear why the authors only described missingness for alcohol and coffee intake. To be responsive to the editor's previous comment please add in a supplemental table the % missing for each dietary variable that was assessed and refer to this table in the methods or result section so that readers can judge for themselves about the potential magnitude of this problem. The very short and vague sentence in the discussion about "informative missingness" should also refer to this table.

R. We have added the proportion of samples used compared to the number of people who have responded to the questionnaire. We have also added relevant reference in the methods and the discussion.

Line 124: “Finally, we have excluded people who reported eating certain foods (e.g. beef) less than once a week due to the very large range of different consumptions which this response corresponds to”

Line 481: “Another source of bias may be due to the missing samples due to either nonresponse or to removal due to the phenotype definition which may induce spurious correlation similarly to sampling bias. Our method however being agnostic to the source of bias, should be able to include these bias and thus select out the SNPs which are associated because of these biases.”
