# Supplementary material for: Using genetic variation to disentangle the complex relationship between food intake and health outcomes
Source: PLoS Genet. 2022 Jun 2;18(6):e1010162. doi: 10.1371/journal.pgen.1010162 (PMC9162356; doi:10.1371/journal.pgen.1010162)
Supplement: Attachment — Submitted filename: Reply to editor.docx [file pgen.1010162.s006.docx]

We would like to thank the editor for the additional comments and we hope that we have answered thoroughly to their doubts. Following a point by point response to the comments.

The authors state that they “have added the proportion of samples used compared to the number of people who have responded to the questionnaire.” It is not clear where this information was added. The table that shows these results need to be specified in the paper and the table needs to be added to the main text or supplement. Furthermore, the authors should add the % missing not only compared to the number of people who have responded to the questionnaire but who was asked to respond to the questionnaire as this is the truly critical denominator to judge missingness.

R. Thank you for pointing this out. We have added this information in Supplementary Table 2. The FFQ and alcohol questionnaires were part of the initial baseline assessment and thus all ~501,000 UK biobank participants have completed it. The numbers now refer to the total number of participants as the denominator.

The authors added “Finally, we have excluded people who reported eating certain foods (e.g. beef) less than once a week due to the very large range of different consumptions which this response corresponds to” It is not clear that this is a good approach. It is very unusual to exclude an entire response group from the analysis as this will limited the range of exposure, which in this case is even not that large as it goes from <1 per week to 0 times per week. There are many good approaches to deal with these type of situations, such as evaluate national dietary assessments to estimate the average intake in this group (<1/wk) and evaluate factors that impact these, such as race, ethnicity, sex, BMI,… so that a value can be assigned within population substrata.

R. We would like to emphasize that there are many ways to perform quality control assessment, however in a GWAS setting, these broad responses should be handled with particular care. The people who fell in this category in some cases were more than half of the total sample; for example, for beef it is 221K out of 501K participants. We could have thus decided to select an arbitrary value to all of them (say once a month) but this would have skewed the distribution closer to 0 or 1 depending on this choice. This is deprecated in GWA studies as it can lead to biases in the effect size estimation.

Another option would have been to, as the editor suggests, impute the values based on other factors such as age, sex, ethnicity, BMI and others. The problem is that imputation quality is poor in the context of moderate to large proportions of missing data (PMID: 21225900). Further, many of these variables that could be used to impute missing data are heritable, and thus conditioning the analyses onto them would have led to collider bias. Given the very high number of people in this category, this could have resulted in false positive genetic associations (Pirastu et al 2021, PMID: 33888908, Day et al 2016 PMID: 26849114). This is particularly problematic in our case where these traits can have causal effects on self-reporting bias and hence such imputation could introduce artificial causal effects and certainly badly calibrated MR estimates. To circumvent this, multiple imputation could have to be performed, which is however computationally unfeasible when running associations on such a large scale.

For these reasons, we decided to prioritise precision rather than power and excluded people for which it was impossible to know food consumption besides its boundaries.

The authors added Line 481: “Another source of bias may be due to the missing samples due to either nonresponse or to removal due to the phenotype definition which may induce spurious correlation similarly to sampling bias. Our method however being agnostic to the source of bias, should be able to include these bias and thus select out the SNPs which are associated because of these biases.”
What evidence exist to make the broad claim that the 2nd sentence is true? It seems that this sentence should be deleted.

R. We agree, this was based on our empirical evidence, but we should not generalize, thus we have deleted the sentence from the discussion.
